# Factors associated with ocular surface epithelial damage in patients with primary Sjögren’s syndrome

**DOI:** 10.1186/s12886-021-01871-0

**Published:** 2021-03-02

**Authors:** Ji Eun Kim, Na Rae Kim, Hee Seung Chin, Kyoung Yul Seo, Tae-im Kim, Ji Won Jung

**Affiliations:** 1 Department of Ophthalmology and Inha Vision Science Laboratory, Inha University School of Medicine, 27, Inhang-Ro, Jung-gu, 22332 Incheon, South Korea; 2 Department of Ophthalmology, Severance Hospital, Yonsei University College of Medicine, Seoul, South Korea

**Keywords:** Dry eye disease, Primary Sjögren's syndrome, Ocular surface epithelial damage, Ocular staining score, Fluorescein tear break up time, Meibomian gland dysfunction

## Abstract

**Background:**

The aim of this study was to evaluate the effects of systemic parameters, laboratory findings, oral parameters, and other ocular surface parameters on ocular surface epithelial damage in patients with primary Sjögren’s syndrome (pSS).

**Methods:**

A total of 82 dry eye disease (DED) patients with pSS were enrolled in this study. Ocular surface epithelial damage was measured by ocular staining score (OSS). Systemic parameters, laboratory findings including serologic markers, oral parameters, and other ocular surface parameters were collected. Other ocular surface parameter assessments such as the Schirmer’s test, fluorescein tear breakup time, meibomian gland examinations, noninvasive keratographic tear film break-up time measurements using the Keratograph® 5 M were performed, and the Ocular Surface Disease Index was determined.

**Results:**

In a multivariate analysis, decreased age and increased duration of pSS were significantly related to increased logarithm-transformed OSS (β = -0.011, *P* = 0.043 and β = 0.003, *P* = 0.008). Among the ocular surface parameters, decreased fluorescein tear breakup time and increased MGD grade were significantly associated with increased logarithm-transformed OSS (β = -0.183, *P* < 0.001 and β = 0.192, *P* = 0.049).

**Conclusions:**

Ocular surface epithelial damage in patients with pSS was associated with young age, long duration of disease, unstable tear film, and decreased meibomian gland function.

## Background

Dry eye disease (DED) is a multifactorial ocular surface disease characterized by a loss of tear film homeostasis [1, 2]. In the 2017 Tear Film and Ocular Surface society (TFOS) Dry Eye Workshop (DEWS) II report, DED was classified into aqueous deficient dry eye (ADDE) and evaporative dry eye. ADDE is categorized into two groups, Sjögren’s syndrome (SS)-related dry eye and non-SS DED [2]. In patients with significant ADDE, 11.6 % had SS: 6.4 % had primary SS (pSS), and 5.2 % had secondary SS [3].

SS is a systemic autoimmune disease, in which T cells and autoantibody producing B cells infiltrate exocrine glands, such as salivary gland and lacrimal gland [4–6]. Th1- and Th17-associated cytokines, IFN-γ, and IL-17, are all associated with increased inflammation and glandular dysfunction, and IL-1 suppress lacrimal acinar secretion [7–9]. This inflammation also involves the extragland, which causes symptoms of pain, myalgia, and inflammation of the joint, vascular system, skin, lungs, and kidneys [10]. When SS occurs by itself, it is referred to as pSS, and when accompanied by another autoimmune disease, it is referred to as secondary SS [11]. The diagnosis of pSS is made in combination with at least 1 symptom of ocular or oral dryness or systemic manifestations as well as signs such as ADDE findings; decreased tear secretion and significant ocular staining score (OSS), the presence of autoantibodies, evidence of reduced salivary gland secretion, and positive findings of minor salivary gland biopsy [12].

In DED, excessive dryness causes tear film hyperosmolarity, which stimulates the production of cytokines from the ocular surface epithelium [13]. This process activates an inflammatory cascade at the ocular surface, which leads to corneal barrier disruption and conjunctival goblet cell dysfunction [13]. The consequent ocular surface epithelial damage can be seen in severe DED and as an indicator of ocular surface epithelial damage, and the OSS has been demonstrated to be an informative marker of disease severity of DED [14]. Also, the OSS showed positive correlations with the expression of inflammatory cytokines in SS related DED, which is an important marker of ocular surface inflammation [13]. Previous few studies have also reported an association between the OSS and not only ocular surface inflammation, but also systemic parameters such as positive serologic findings [15, 16].

**Fig. 1:**
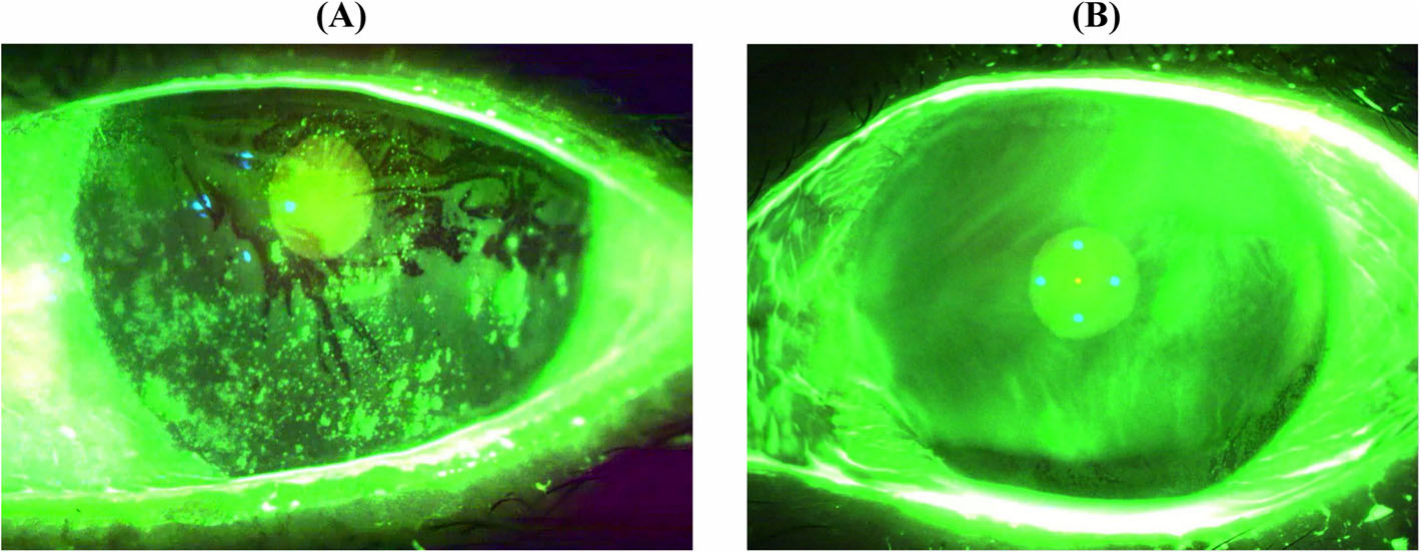
The K5M images of ocular surface staining in two dry eye patients with primary Sjögren’s syndrome (pSS). **a** Severe ocular surface staining in a 60-year-old woman with pSS. **b** Mild ocular surface staining in a 59-year-old woman with pSS

The factors that affect ocular surface epithelial damage in the patients with pSS (Fig. 1a, b) have not been well studied yet. Furthermore, this type of damage is important in determining optimal treatment strategies for DED patients with pSS. Therefore, the purpose of our study was to evaluate the effects of systemic parameters, laboratory findings, oral parameters, and other ocular surface parameters on ocular surface epithelial damage in patients with pSS by a multivariate analysis.

## Methods

### Subjects

The study was approved by the Institutional Review Board (IRB) of Inha University Hospital and it was complied with the tenets of the Declaration of Helsinki. No written informed consent was required as data was retrospectively collected from chart review, as approved by the reference IRB.

This retrospective chart review of patients with SS among outpatients who visited for management of dry eye symptoms from March 2015 to July 2019 in the Department of Ophthalmology at Inha University Hospital. One hundred thirty-five SS patients with recorded ocular surface parameters (including the OSS) and checkable medical chart were analyzed, and 73 patients who were excluded based on the pSS criteria. A total of 82 patients with DED who were diagnosed with pSS were enrolled in this study. The American College of Rheumatology/European League against Rheumatism classification criteria for rheumatoid arthritis (ACR/EULAR) was used to diagnose pSS [17], which was defined as a score ≥ 4 with at least 1 symptom of ocular or oral dryness or the presence of systemic manifestations of SS. We excluded patients younger than 20 years of age as well as those with histories of ocular surgery within 6 months, ocular injury, or other ocular diseases.

### Outcome measures

The clinical variables, presence of systemic disease, and previous medical history were investigated. The age was defined as age at the time of examination of OSS, and the duration of pSS was defined as the period from the diagnosis of pSS to the examination of OSS. We assessed the current medication use at the time of examination of OSS, at least 3 months of use were included. Oral medications such as pilocarpine, hydroxychloroquine, methotrexate, cyclosporine, and steroid for SS, and topical medications such as cyclosporine and steroid for DED were included. The duration of the topical cyclosporine use was 13.2 months (range, 6–25 months) and topical steroid use was 11.3 months (range, 3–18 months).

Laboratory parameters included white blood cell, erythrocyte sedimentation rate, C-reactive protein, complement C3, complement C4, and immunoglobulin G. The positivity of antibodies associated with connective tissue diseases, such as anti-Sjögren’s-syndrome-related antigen A(SSA)/Ro, anti- Sjögren’s-syndrome-related antigen B(SSB)/La, antinuclear antibody (ANA), and rheumatoid factor (RF), were assessed. The positive ranges of antibodies are as follows: anti-SSA/Ro antibody > 10 U/mL, anti-SSB/La antibody > 10 U/mL, ANA ≥ 1:80, and RF > 14.0 IU/mL. All laboratory parameters were measured at the Inha University Hospital laboratory medicine department, and the laboratory findings closest to the time of examination of OSS at Inha University hospital ophthalmology outpatient clinic were used. The average time interval between the laboratory findings and examination of OSS was 6.1 ± 6.6 months.

Ocular surface parameters were performed as follows and data were obtained from the right eye unless the right eye was excluded from the study, in which case data were collected from the left eye. (1) OSS according to the National Eye Institute/Industry Workshop scale [17–20], which was graded from 0 to 3 for each of the five areas on cornea and each of the six areas on conjunctiva. A total score from 0 to 33 based on the pattern of fluorescein staining observed under the slit lamp. (2) Subjective symptoms were graded on a numerical scale from 0 to 4, according to the validated 12-item ocular surface disease index (OSDI) questionnaire. The total OSDI, ranging from 0 to 100, was calculated using the following formula: OSDI = (sum of scores for all questions answered × 100) / (total number of answered questions × 4) [18, 20–22]. (3) Schirmer’s test I was performed without topical anesthesia by placing a Schirmer strip in the mid-lateral portion of the lower fornix. The amount of wetting was recorded after 5 min, and the patients were asked to keep their eyes lightly closed during the test. (4) Fluorescein break-up time (FBUT) was measured through application of a single fluorescein strip (Haag-Streit, Koeniz, Switzerland) to the inferior palpebral conjunctiva after instilling a drop of normal saline. The mean time for three results was recorded. (5) The lid margins and meibomian glands were checked for lid margin abnormalities, gland expression, and meibum quality, as previously described [20, 21, 23–27]. Lid margin abnormalities were scored as 0 (absent) or 1 (present) for the following parameters: vascular engorgement, plugged meibomian gland orifices, anterior or posterior displacement of the mucocutaneous junction, and irregularity of the lid margin [21, 23–25, 27]. The presence of an inflamed lid margin was checked. The degree of meibomian gland expressibility using firm digital pressure applied on five glands of the central third of the lower lid was graded as follows: grade 0, all five glands expressible; grade 1, three to four glands expressible; grade 2, one to two glands expressible; and grade 3, no glands expressible [24, 26, 27]. The meibum quality over eight lower lid glands was graded as follows: grade 0, clear; grade 1, cloudy; grade 2, cloudy with granular debris; and grade 3, thick (texture similar to toothpaste). Each of the eight glands of the lower eyelid was graded on a scale from 0 to 3. The scores of the eight glands were summed to obtain a total score (range, 0–24) [21, 24, 27]. The meibomian gland dysfunction (MGD) stage was determined based on the three lid parameters: MGD stage 1, minimally altered expressibility (grade 1) and secretion quality (2 ≤ grade < 4); MGD stage 2, scattered lid margin features, mildly altered expressibility (grade 1) and secretion quality (4 ≤ grade < 8); MGD stage 3, lid margin features of plugging, vascularity, moderately altered expressibility (grade 2) and secretion quality (8 ≤ grade < 13); MGD stage 4, lid margin features of dropout, displacement, severely altered expressibility (grade 3), and secretion quality (grade ≥ 13) [23, 27]. (6) Tear film assessment with the “TF-Scan, Non-Invasive Keratograph® Break-Up Time (NIKBUT) mode” was conducted when the subjects were instructed to blink three or four times and then keep their eyes open for as long as possible. Irregularities on the image indicated instability or breakup of the tear film and, at the same time, a video was recorded. The device provided a representation of tear film break up over time, including a tear film map showing the location and size of the tear film-break regions, as well as the first break-up time (NIKBUT-first) and the average break-up time (NIKBUT-average; the mean of all tear film break-ups occurring over the entire cornea), as previously described [20, 28, 29].

Oral parameters included subjective oral symptom score, non-stimulated whole salivary flow (NSWSF), and positivity of a minor salivary gland biopsy. The subjective oral symptom score was based on the 2002 American-European Consensus Group (AECG) criteria, and three questions were summed to obtain a total score (range, 0–3): (1) symptoms of dry mouth for at least 3 months, (2) recurrent or persistently swollen salivary glands, and (3) need for liquids to swallow dry foods [17]. NSWSF reflects the basal flow from all glands. Patients must swallow any residual saliva before starting the procedure and allow all saliva to accumulate in the mouth and spit it every minute. Saliva was collected for 15 minutes and the measured volume was expressed in mL/min. For the SS criteria, a value ≤ 0.1 mL/min was considered abnormal [17, 30]. Minor salivary gland biopsy positivity was determined based on the AECG criteria, which was defined as a focus score of 1 or more. Focus was defined as a dense aggregate of 50 or more lymphocytes in a 4-mm^2^ area of the glandular tissue [17].

### Statistical analyses

Statistical analyses were performed using SPSS for Windows (version 20.0; SPSS Inc., Chicago, IL, USA). The OSS was logarithm-transformed to be approximately normally distributed. Univariate and multivariate linear regression analyses were computed to evaluate the impact of systemic and laboratory parameters, oral parameters, and other ocular surface parameters on the logarithm-transformed OSS. For the multivariate linear regression analysis, age, sex, and other variables with *P* value less than 0.1 in the univariate model were included. In all tests, a *P* value less than 0.05 was considered significant.

## Results

A total of 82 DED patients with pSS were included in this study. Table 1 shows the clinical variables, presence of systemic disease, and previous medical history of 82 patients. The mean age was 53.8 years old (range, 24–80 years), and 78 patients (95.1 %) were women. The mean duration of pSS was 39.4 months (range, 1–185 months). The laboratory findings and oral parameters of all 82 patients are summarized in Table 2: 77.2 % of the patients were positive for anti-SSA/Ro, 38.6 % were positive for anti-SSB/La, and the proportion of both positive were 41.5 %. 63.4 % were positive for ANA, and 43.8 % were positive for RF. The ocular surface parameters and keratographic parameters of the 82 patients are summarized in Table 3: the mean OSS was 7.65 ± 5.06 (range, 0–26).

**Table 1:** The systemic parameters and univariate analysis of these parameters on logarithm transformed ocular staining score

Variables	DED related to pSS (***n***=82)	Univariate analysis	
		Beta (SE)	***P***-value
Age (y)	53.8 ± 11.2 (24-80)	0.009 (0.006)	0.136
Sex, n (%)			
Male	4 (4.9%)	1 (Ref)	
Female	78 (95.1%)	0.005 (0.309)	0.988
Duration of disease (m)	39.4 ± 45.4 (1-185)	0.003 (0.001)	**0.029**
Systemic disease, n (%)			
No history	61 (74.4%)	1 (Ref)	
Hypertension	16 (19.5%)	0.144 (0.167)	0.389
Diabetes mellitus	3 (3.7%)	0.250 (0.353)	0.482
Ocular surgery history, n (%)			
No history	69 (84.1%)	1 (Ref)	
Refractive surgery	6 (7.3%)	-0.356 (0.252)	0.162
Pharmacological history, n (%)			
Oral Pilocarpine	16 (19.5%)	-0.024 (0.134)	0.857
Oral Hydroxychloroquine	3 (3.7%)	-0.134 (0.147)	0.365
Oral Methotrexate	15 (18.3%)	-0.029 (0.172)	0.864
Oral Cyclosporine	3 (3.7%)	0.413 (0.351)	0.243
Oral Steroid	14 (17.1%)	0.204 (0.175)	0.246
Topical Cyclosporine	3 (3.7%)	0.551 (0.185)	**0.004**
Topical Steroid	11 (13.4%)	0.218 (0.175)	0.217

*DED* dry eye disease, *pSS* primary Sjögren’s syndrome, *SE* standard error

**Table 2:** Laboratory findings, oral parameters, and univariate analysis of these parameters on logarithm-transformed ocular staining score

Variables	Mean ± SD	Univariate analysis	
		Beta (SE)	***P***-value
Laboratory findings			
White blood cell (/μL)	5122.56 ± 1496.99	0.000 (0.000)	0.350
Erythrocyte sedimentation rate (mm/h)	17.09 ± 13.46	0.006 (0.005)	0.262
C-reactive protein (mg/dL)	0.14 ± 0.33	0.138 (0.203)	0.498
Complement C3 (mg/dL)	105.68 ± 19.71 (68^a^)	0.003 (0.004)	0.471
Complement C4 (mg/dL)	21.79 ± 8.32 (68^a^)	0.005 (0.009)	0.563
Immunoglobulin G (mg/dL)	1412.61 ± 407.95 (38^a^)	0.000 (0.000)	0.124
Positive serology % (n)			
Anti-SSA/Ro antibody	77.2 (61/79^a^)	0.274 (0.157)	0.086
Anti-SSB/La antibody	38.6 (27/70^a^)	0.504 (0.140)	**0.001**
Antinuclear antibody, (≥1: 80)	63.4 (52/82^a^)	0.235 (0.135)	0.087
Rheumatoid factor	43.8 (35/80^a^)	0.315 (0.132)	**0.020**
Oral parameters			
Subjective oral score (0-3)	1.58 ± 0.71	0.258 (0.093)	**0.007**
Non-stimulated whole salivary flow (mL/min)	0.09 ± 0.09 (79^a^)	-0.122 (0.047)	**0.011**
Positive results of salivary gland biopsy, % (n)	73.7 (14/19^a^)	0.119 (0.293)	0.688

^a^Represented the actual number of patients who underwent the test

*SD* standard deviation, *SSA* Sjögren's-syndrome-related antigen A, *SSB* Sjögren's-syndrome-related antigen B; *SE* standard error

**Table 3:** Ocular surface parameters and univariate analysis of these parameters on logarithm-transformed ocular staining score

Variables	Mean ± SD	Univariate analysis	
		Beta (SE)	***P***-value
Ocular surface parameters			
Ocular staining score (0-33), NEI scale	7.65 ± 5.06		
Subjective score (OSDI)	41.63 ± 22.88	0.004 (0.003)	0.179
Schirmer’s test I value (mm)	7.01 ± 6.83	-0.038 (0.008)	**<0.001**
FBUT (seconds)	2.30 ± 1.67	-0.230 (0.026)	**<0.001**
MGD grade (0–4)	1.95 ± 0.74	0.432 (0.076)	**<0.001**
Parameters using K5M			
NIKBUT-first (seconds)	4.31 ± 1.73	-0.069 (0.035)	0.054
NIKBUT-average (seconds)	8.20 ± 3.12	-0.026 (0.020)	0.193

*K5M* Keratograph®5M, *FBUT* fluorescein break-up time, *MGD* meibomian gland dysfunction, *NEI* National Eye Institute, *NIKBUT* non-invasive Keratograph® break-up time, *OSDI* ocular surface disease index, *SE* standard error

The results of univariate linear regression analysis are summarized in Tables 1, 2 and 3. The duration of pSS, topical cyclosporine history, anti-SSB/La antibody, RF, MGD stage, and subjective oral score were positively associated with logarithm-transformed OSS. FBUT, Schirmer’s test-I value, NSWSF were negatively associated with logarithm-transformed OSS (all *P* < 0.050).

In the multivariate linear regression analysis, age, sex, and statistically significant or borderline significant (*P* value less than 0.1) variables were included. After adjusting for other factors, decreased age and increased duration of pSS were significantly related to increased logarithm-transformed OSS. (β = -0.011, *P* = 0.043 for age; β = 0.003, *P* = 0.008 for duration of pSS). After adjusting for other factors, positive serology or oral parameters was not related to OSS in our study. Among the ocular surface parameters, decreased FBUT and increased MGD grade were significantly associated with increased logarithm-transformed OSS (β = -0.183, *P* < 0.001 for FBUT; β = 0.192, *P* = 0.049 for MGD grade) (Table 4).

**Table 4:** Univariate and multivariate analysis of variables on logarithm-transformed ocular staining score

Variables	Univariate analysis		Multivariate analysis	
	Beta (SE)	***P***-value	Beta (SE)	***P***-value
Age (y)	0.009 (0.006)	0.136	-0.011 (0.005)	**0.043**
Sex, n (%)				
Male	1 (Ref)		1 (Ref)	
Female	0.005 (0.309)	0.988	-0.097 (0.250)	0.699
Duration of disease (m)	0.003 (0.001)	0.029	0.003 (0.001)	**0.008**
Pharmacological history, n (%)				
Topical Cyclosporine	0.551 (0.185)	0.004	-0.008 (0.161)	0.962
Positive serology % (n)				
Anti-SSA/Ro antibody	0.274 (0.157)	0.086	-0.082 (0.144)	0.572
Anti-SSB/La antibody	0.504 (0.140)	0.001	0.103 (0.142)	0.476
Antinuclear antibody, (≥1: 80)	0.235 (0.135)	0.087	0.191 (0.122)	0.123
Rheumatoid factor	0.315 (0.132)	0.020	0.096 (0.114)	0.404
Ocular surface parameters				
Schirmer’s test I value (mm)	-0.038 (0.008)	<0.001	-0.012 (0.007)	0.105
FBUT (seconds)	-0.230 (0.026)	<0.001	-0.183 (0.033)	**<0.001**
MGD grade (0–4)	0.432 (0.076)	<0.001	0.192 (0.095)	**0.049**
Parameters using K5M				
NIKBUT-first (seconds)	-0.069 (0.035)	0.054	0.053 (0.030)	0.084
Oral parameters				
Subjective oral score (0–3)	0.258 (0.093)	0.007	0.074 (0.067)	0.279
NSWSF (mL/min)	-0.122 (0.047)	0.011	-0.025 (0.045)	0.578

*K5M* Keratograph®5M, *FBUT* fluorescein break-up time, *MGD* meibomian gland dysfunction, *NEI* National Eye Institute, *NIKBUT* non-invasive Keratograph® break-up time, *NSWSF* non-stimulated whole salivary flow, *SE* standard error, *SSA* Sjögren's-syndrome-related antigen A, *SSB* Sjögren's-syndrome-related antigen B

## Discussion

The aim of this study was to identify the factors associated with ocular surface epithelial damage in patients with pSS. We evaluated the effects of systemic parameters, laboratory findings, oral parameters, and other ocular surface parameters on ocular surface epithelial damage in patients with pSS. Ocular surface epithelial damage was measured by OSS, which is sum of corneal and conjunctival staining score. The OSS is included in the criteria for pSS with Schirmer’s test, and it is an informative marker for the severity of DED [12, 14]. Because ocular surface epithelial damage could affect visual disturbance and indicates the presence of ocular surface inflammation, the OSS parameter is important for determining treatment for DED patients with pSS [13, 16, 31].

Previous studies have reported that an association between dry eye and systemic parameters, or extraocular variables [11, 16, 32–35]. They used the symptoms of ocular dryness, tear osmolarity, or severe DED severity, as indicators of DED. Similar to our study, few studies used the OSS as the main outcome variable, but only reported correlations with laboratory findings, such as positive serologic findings [15, 16]. Therefore, we focused on the OSS as the main outcome and analyzed its independent association with systemic parameters, laboratory findings, oral parameters, and other ocular surface parameters after adjusting other variables.

Among the systemic parameters, age and duration of pSS were significant factors that influenced OSS in the multivariate analysis of our study. A decrease in age was significantly related to an increase in OSS after adjusting for other factors. Although the previous studies of ocular parameters in patients with pSS have reported a correlation with age, they mostly have found a correlation with onset age of pSS or with ocular symptoms other than OSS [32–36]. In their study, the age was negatively related to ocular dryness, indicating older patients with severe disease tended to be less sensitive [32].

 In another study, young-onset SS (age at diagnosis < 35 years) correlation with xerostomia (*P* = 0.008), abnormality of Schirmer’s test and/or Rose Bengal staining (*P* = 0.03), positivity of anti-Ro/SS-A antibodies (*P* < 0.001), low complement C3 (*P* = 0.018) and low complement C4 levels (*P* = 0.017), compare to age at diagnosis > 35 years [33].

An increase of the duration of pSS was significantly related to an increase of OSS in the current study. A previous study with a large cohort study of patients with pSS in Spain also reported a correlation between duration of pSS and ocular involvement. Patients who had longer duration of pSS (more than 10 years) showed a higher prevalence of xerophthalmia, abnormality of Schirmer’s test and/or Rose Bengal staining and other systemic involvements (parotid enlargement, lung involvement, vasculitis, or peripheral neuropathy), laboratory findings (positivity of anti-SSA/Ro antibody, anti-SSB/La antibody, and low complement C4 level) in a univariate analysis. But in a multivariate analysis, there were no significant correlations with xerophthalmia and abnormality of Schirmer’s test and/or Rose Bengal staining that influence duration of pSS after adjusting for other factors [33]. According to this our study, patients with young age and long duration of pSS have higher risk of ocular surface damage; therefore, more careful monitor of DED is needed in those patients with pSS.

In our study, among the medical history, topical cyclosporine was associated with increased OSS in univariate analysis. This may have been due to the fact that topical cyclosporine was prescribed as treatment in order to improve the OSS in patients with severe ocular surface epithelial damage. However, after adjusting for other factors, no significant association was found in the multivariate analysis.

Of the laboratory findings in our study, positivity of anti-SSB/La antibody and RF were significant variables, and positivity of anti-SSA/Ro antibody and ANA were borderline significant variables in the univariate analysis. This result was similar with previous studies, that reported a correlation between the ocular surface parameters and serologic markers in patients with pSS [3, 15, 16]. Serum RF, and ANA levels correlated with conjunctival staining score and total OSS in patients with pSS [15]. Furthermore, serum anti-Ro/SSA and anti-La/SSB antibodies were significantly related with clinical severity of keratoconjunctivitis sicca based on the Oxford OSS scheme in patients with pSS [16]. These studies suggested that these parameters could be considered as prognostic factors for predicting the severity and prognosis of DED in patients with pSS [15, 16]. However, in our multivariate analysis, there were no significant laboratory findings that influenced OSS after adjusting for other factors. This was the first multivariate analysis study of association between serologic markers and OSS, and the results might differ compare to previous studies that performed a univariate analysis.

Among the oral parameters, positive subjective oral score and NSWSF were significantly related with OSS in the univariate analysis; however, after adjusting for other factors, no significant correlation was found. In a recent rheumatologic study, only the presence of inflammatory joint involvement among other systemic manifestations was associated with severe/very severe DED in patients with pSS (odds ratio, 2.079) [35]. Similarly, a multivariate analysis was performed in this study, thereby showing that there were no associations between other laboratory findings or oral parameters and DED severity.

Among the ocular surface parameters, FBUT and MGD were significant factors that influenced OSS in the multivariate analysis of our study. An increase in FBUT was significantly related to an increase in OSS after adjusting for other factors. The results of the Schirmer’s test were significantly related with OSS in the univariate analysis, but no significant correlation was found after adjusting for other factors. According to previous study of patients with DED and ocular surface disease, FBUT is minimally invasive test and is a more reliable than the Schirmer’s test. Furthermore, FBUT is strongly correlated with other ocular tests such as OSS [37]. From these results, FBUT reflected the ocular surface epithelial damage better than the Schirmer’s test even in patients with pSS.

An increase in MGD grade was significantly related to an increase in OSS after adjusting for other factors. A previous study on MGD in patients with pSS reported that changes in the meibomian gland induced an increase of tear evaporation and subsequent worsening of the ocular surface desiccation [38]. Furthermore, in another study, morphological and functional features of the meibomian gland correlated with other ocular surface parameters and disease severity in ADDE, such as pSS and graft-versus-host disease [39]. According to this result, more attention is needed in pSS patients with low FBUT and severe MGD, which could cause more ocular surface epithelial damage.

There were several limitations in this study. First, the retrospective of the study might lead to unexpected various bias. Second, only a small number of patients were enrolled. Third, some of the parameters were not assessed in some of the patients. Fourth, some systemic manifestations, such as inflammatory joint involvement, were not evaluated in this study. Because of these limitations, it is necessary to conduct a prospective study in the future. However, our study is meaningful because it is the first multivariate regression analysis of factors associated with ocular surface epithelial damage in patients with pSS.

## Conclusions

In conclusion, ocular surface epithelial damage in patients with pSS was associated with young age, long duration of disease, unstable tear film, and decreased meibomian gland function.

## Data Availability

Data sets used in this study are available from the contributing author upon reasonable request.

## Abbreviations

SS: Sjögren’s syndrome
OSS: Ocular surface staining score
pSS: Primary Sjögren’s syndrome
DED: Dry eye disease
MGD: Meibomian gland dysfunction
FBUT: Fluorescein tear break-up time
NSWSF: Non-stimulated whole salivary flow
TFOS: Tear Film and Ocular Surface society
DEWS: Dry Eye Workshop
ADDE: Aqueous deficient dry eye
Th: T helper cell
IFN: Interferon
IL: Interleukin
NIKBUT: Non-Invasive Keratograph® Break-Up Time
SSA: Sjögren's-syndrome-related antigen A
SSB: Sjögren's-syndrome-related antigen B
ANA: Antinuclear antibody
RF: Rheumatoid factor
NEI: National Eye Institute
OSDI: Ocular Surface Disease Index
K5M: Keratograph®5M
GVHD: Graft-versus-host disease
ACR/EULAR: American College of Rheumatology/European League against Rheumatism classification criteria for rheumatoid arthritis
AECG: American-European Consensus Group
